# High tumor burden score indicated the unfavorable prognosis in patients with hepatocellular carcinoma: A meta-analysis

**DOI:** 10.1371/journal.pone.0308570

**Published:** 2024-08-08

**Authors:** Wangbin Ma, Rongqiang Liu, Jianguo Wang, Li Liu, Zhendong Qiu, Jia Yu, Weixing Wang

**Affiliations:** Department of Hepatobiliary Surgery, Renmin Hospital of Wuhan University, Wuhan, Hubei Province, China; Helwan University, EGYPT

## Abstract

**Background:**

Tumor burden score (TBS) based on maximum tumor diameter and number has been shown to correlate with prognosis in patients with hepatocellular carcinoma (HCC). Nevertheless, the results are conflicting. Hence, we conducted a meta-analysis to analyze the association between TBS and survival outcomes of HCC patients.

**Methods:**

A comprehensively search of the databases including PubMed, Embase and Web of Science was performed to retrieve studies satisfying the inclusion criteria until August 31, 2023. The hazard ratios (HRs) and 95% confidence intervals (CIs) were calculated. All the data analyses were carried out by STATA 12.0.

**Results:**

10 retrospective studies containing 25073 patients were incorporated in the study. The results demonstrated that high TBS was markedly association with poor overall survival (OS) (HR: 1.79, 95% CI: 1.45–2.23) and relapse-free survival / progression-free survival(RFS/PFS) (HR: 1.71; 95% CI: 1.42–2.07). Subgroup analysis showed that the prognostic value of TBS in HCC was not affected by any subgroup.

**Conclusions:**

TBS may be an efficient prognostic index in HCC patients.

## Introduction

Hepatocellular carcinoma (HCC) is one of the most common malignancies and the third primary reason of tumor-concerned death worldwide [1]. There were about 906,000 cases of liver tumor newly diagnosed and 830,000 cases of liver tumor-concerned deaths worldwide in 2020 [2]. In the United States, epidemiological data predicts that the incidence of HCC will continue to increase [3]. Hazardous elements of HCC contain hepatitis virus infection, aflatoxin, alcoholism and non-alcoholic fatty liver [4]. For early-stage patients, potentially curative treatments such as tumor resection, locoregional ablation and liver transplantation are considered the optimal therapeutic candidates [5]. However, even with efficient remedy, the 5-year survival outcome of patients with progressive liver tumor is still under 10% [6]. Tumor burden is an essential element impacting the prognosis of HCC patients. However, simple comparison of tumor size or tumor number cannot accurately predict the survival of HCC patients.

Tumor burden score (TBS) based on tumor maximum diameter and number was firstly proposed by Sasaki et al as a tool for predicting survival outcome in patients with colorectal liver metastasis (CRLM) after resection [7]. Sasaki et al further confirmed that TBS was a better predictor of prognosis in patients with CRLM compared to traditional measures such as tumor size and numbers [8]. Subsequently, a prospective investigation revealed that TBS could effectively forecast recurrence and prognosis in HCC and had the better adaptation in comparison to other continuous variables [9]. TBS also could stratify patients connected with 5-year survival rate after resection of multinodular HCC beyond milan standard and multinodular HCC patients with low TBS could undergo radical hepatectomy [10]. The prognostic value of TBS was validated in different liver cancer studies [11–15]. However, their conclusions were not entirely consistent.

In the study, we carried out a meta-analysis to synthetically analyze the prognostic significance of TBS in patients with HCC.

## Materials and methods

### Search strategy

We implemented a comprehensive search of the studies investigating the prognostic value of TBS in patients with HCC in the PubMed, Embase, and Web of Science databases using the search terms: “tumor burden score” AND “hepatocellular carcinoma” OR “hepatic carcinoma” OR “hepatoma” OR “liver cancer” OR “HCC”. Search strategy was presented in S1 Table. The search deadline was August 31, 2023. Additionally, we examined the references of the incorporated literature to ensure a thorough search. The present meta-analysis was conducted according to the PRISMA statement (S1 Checklist and S1 Fig).

### Study selection

The document retrieval process was independently implemented by three researchers (Wangbin Ma, Rongqiang Liu and Jianguo Wang). Differences were resolved through discussion. Articles that met the following requirements were considered eligible: (1) study evaluated the prognostic outcomes of TBS in HCC patients; (2) adequate data to compute hazard ratios (HRs) and 95% confidence intervals (CIs). (3) articles were published in English or non-English. (4)the treatment methods of HCC patients, including surgery, immunotherapy, targeted therapy, radiotherapy and chemotherapy and so on, were described. Exclusion criteria: (1) inadequate data studies; (2) case abstracts, comment, reviews, letters, and duplicated studies. (3) cell and animal studies.

### Data extraction and quality evaluation

Data was extracted by two researchers (Wangbin Ma and Rongqiang Liu). Any disagreement was resolved to achieve a consensus by the third investigator (Jianguo Wang). A standardized data collection table was applied to extract the following information: the author’s name, year of publication, study type, country, sample size, survival outcomes, analysis type, source of HR, treatment method and data source. To prioritize the most valuable outcomes, the authors gave priority for multivariate analyses. The quality of the incorporated researches was reviewed with the Newcastle-Ottawa Quality Assessment Scale (NOS) [16].

### Data analysis

The correlation between TBS and survival outcomes in HCC patients was assessed using a forest plot. Heterogeneity was assessed by the higgins I-squared statistic [17]. P value <0.05 or I^2^>50% indicated remarkable heterogeneity, and the random-effects model was applied. In addition, the fixed-effects model was utilized. We conducted subgroup analysis and meta-regression to investigate sources of heterogeneity. Sensitivity analysis was performed to assess the consistency of the consequences. Publication bias was analyzed with Begg’s and Egger’s tests [18, 19]. If publication bias existed, trim and fill method was used to determine the reliability of the meta-analysis [20]. All statistical analyses were operated with STATA 12.0 (Stata Corporation, College Station, TX, USA). P value <0.05 represented statistical significance.

## Results

### Search outcomes

**Fig 1:**
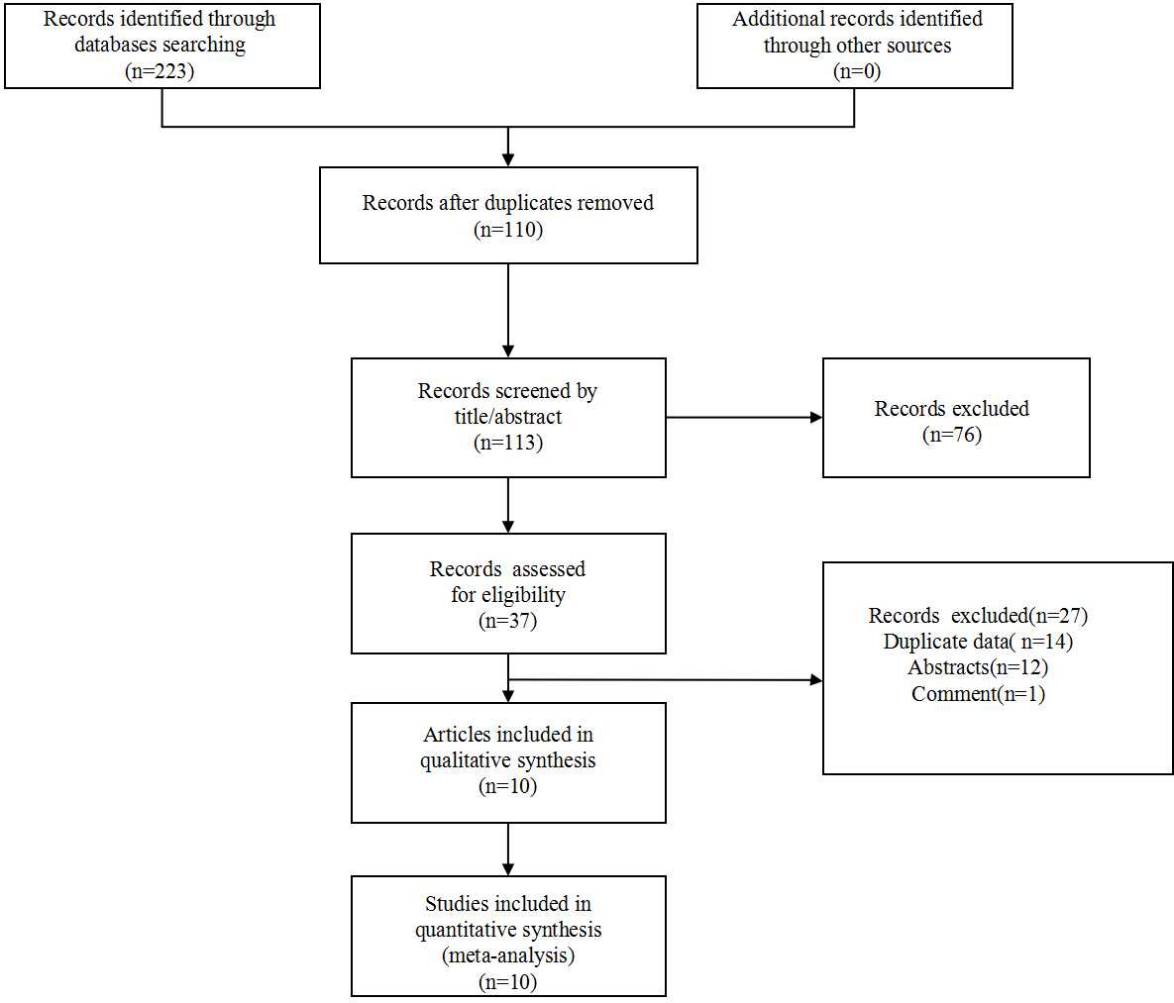
Flow chart of the literature search.

223 articles were incipiently retrieved through a search of the designated databases. After removing 110 duplicate documents, 113 articles remained. After filtering the title and abstract, 27 articles were eliminated. After reviewing the entire content of the publications, 10 articles published between 2018 and 2023 were incorporated in the final analysis [9, 11–15, 21–24]. The flow chart was presented in Fig 1.

### Study characteristics

10 articles included meta-analysis were retrospective studies. 6 studies were from multi-center institution and 4 studies were conducted in single-center institution. The amount of enrolled patients was 25073. 8 studies reported overall survival (OS) data and 5 studies displayed relapse-free survival/progression-free survival data(RFS/PFS). The NOS score of each study ranged from 6 to 8. Basic information for each study was displayed in Table 1.

**Table 1:** Basic information of included studies.

Study	Year	Country	Study type	Sample size	Analysis type	Survival analysis	Source of HR	NOS score	Treatment methods	Data source
Moris	2020	America	Retrospective	12578	Multivariate	OS,RFS	Reported	8	Surgery	Multiple-center
Müller	2022	Germany	Retrospective	849	Multivariate	OS	Reported	7	TACE	Multiple-center
Vitale	2018	Italy	Retrospective	4759	Multivariate	OS	Reported	7	Mixed	Multiple-center
Wang	2022	China	Retrospective	217	Multivariate	RFS	Reported	6	Surgery	Single-center
Moazzam	2022	America	Retrospective	1994	Multivariate	RFS	Reported	6	Surgery	Multiple-center
Li	2023	China	Retrospective	342	Multivariate	OS,RFS	Reported	7	Surgery	Single-center
Yang	2023	China	Retrospective	378	Multivariate	OS,PFS	Reported	7	Immunotherapy	Multiple-center
Yen	2023	China	Retrospective	623	Multivariate	OS	Reported	7	Surgery	Single-center
Ho	2022	China	Retrospective	1898	Multivariate	OS	Reported	7	Mixed	Single-center
Lima	2023	America	Retrospective	1435	Multivariate	OS	Reported	7	Surgery	Multiple-center

**Abbreviation**: TACE, transcatheter arterial chemoembolization; OS, overall survival; RFS, recurrent-free survival; PFS, progression-free survival; NOS, Newcastle-Ottawa Quality Assessment Scale.

### Association between high TBS and OS

**Fig 2:**
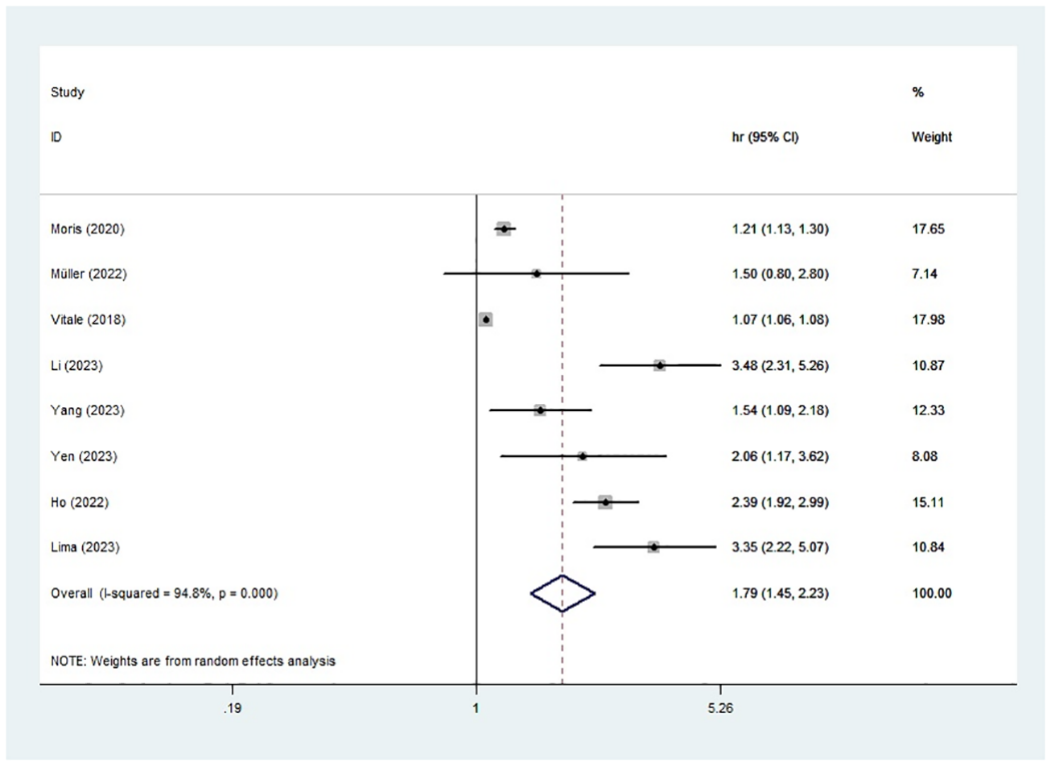
Forest plot of the association between high TBS and OS.

A meta-analysis was carried out by utilizing a random-effects model due to significant heterogeneity (I^2^ = 94.8%). The results indicated a significant correlation between high TBS and inferior OS (HR: 1.79, 95% CI: 1.45–2.23) (Fig 2).

### Subgroup analysis and meta-regression for OS

**Fig 3:**
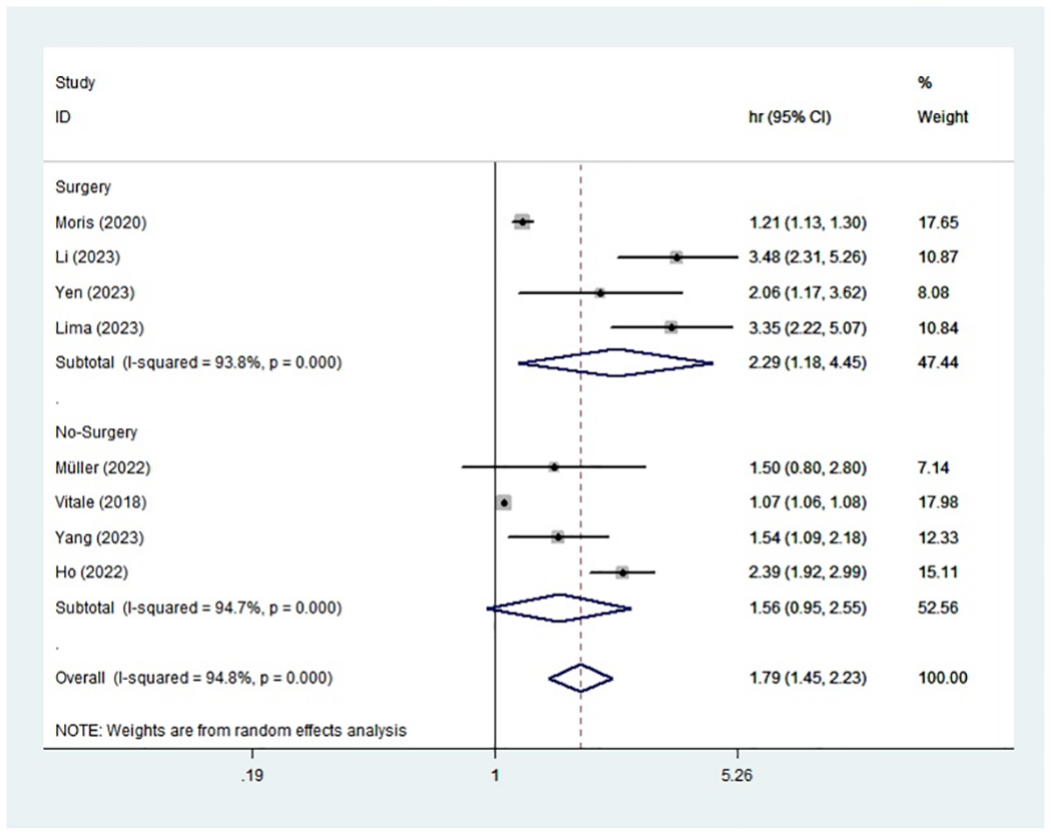
Forest plot of subgroup analysis based on treatment methods.

To investigate the sources of heterogeneity, we conducted subgroup analysis and meta-regression on the basis of treatment method, sample size, publication year and data source (Table 2). The outcomes portrayed that high TBS indicated worse OS in HCC patients after hepatectomy based on the subgroup of treatment method(HR:2.29,95%CI:1.18–4.45) (Fig 3). In the subgroup of year>2021 (HR:2.46, 95%CI:1.61–3.78) or year<2021 (HR:1.40, 95%CI:1.13–1.74), high TBS was associated with an adverse prognosis. In terms of sample size, whether the sample size was greater than 1000 (HR:1.62, 95%CI:1.27–2.07) or less than 1000 (HR:2.05, 95%CI:1.33–3.14), high TBS showed the unfavorable prognosis. Moreover, the subgroup of multiple-center (HR:1.39, 95%CI:1.51–1.66) or single-center (HR:2.54, 95%CI:2.11–3.06) also displayed that HCC patients with high TBS had adverse survival outcome. We also spotted that data source may be the source of heterogeneity using the meta-regression (P = 0.043).

**Table 2:** Subgroups analysis and meta-regression for overall survival.

Variables	No. of studies	Estimate HR (95%)	P value	Heterogeneity		Meta-regression		
				I^2^ (%)	P value	Tau^2^	Adj R^2^ (%)	P value
**Treatment method**						0.1858	-5.70	0.489
Surgery	4	2.29(1.18–4.45)	0.015	93.8	<0.01			
Non- surgery	4	1.56(0.95–2.55)	0.08	94.7	<0.01			
**Year**						0.1893	-7.69	0.827
>2021	4	2.46(1.61–3.78)	<0.01	75.5	0.007			
<2021	4	1.40(1.13–1.74)	0.002	95.3	<0.01			
**Sample size**						0.2042	-11.31	0.658
>1000	4	1.62(1.27–2.07)	<0.01	96.8	<0.01			
<1000	4	2.05(1.33–3.14)	0.001	69.8	0.019			
**Data source**						0.03	71.46	0.043
Multiple-center	5	1.39(1.51–1.66)	0.003	91.5	<0.01			
Single-center	3	2.54(2.11–3.06)	<0.01	34.3	0.218			

### Association between high TBS and RFS/PFS

**Fig 4:**
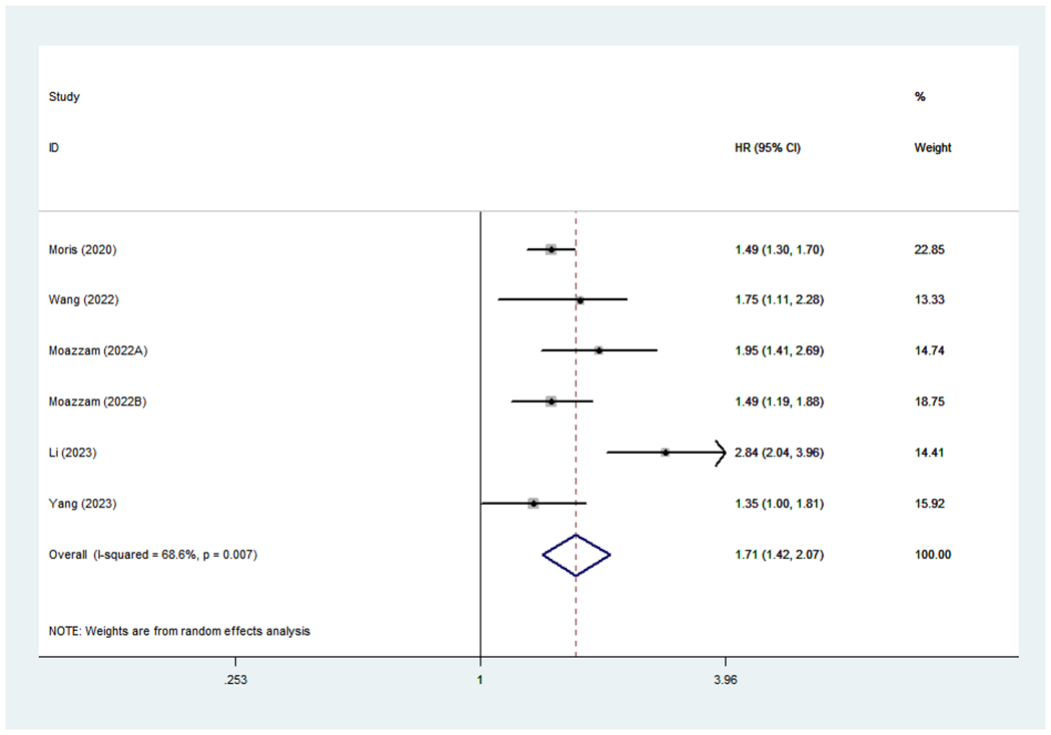
Forest plot of the association between high TBS and RFS/PFS.

6 studies utilized RFS/PFS to probe the correlation between TBS and prognostic outcome of HCC patients. Due to the obvious heterogeneity, we adopted the random-effects model (I^2^ = 68.6%). Meta-analysis suggested that high TBS was significantly connected with inferior RFS/PFS (HR: 1.71; 95% CI: 1.42–2.07) (Fig 4).

We also performed subgroup analysis and meta-regression for RFS/PFS (Table 3). The results of all subgroups showed that high TBS predicted poor RFS/PFS. Subgroup analysis also suggested that sample size and data source may be sources of heterogeneity.

**Table 3:** Subgroups analysis for RFS/PFS.

Variables	No. of studies	Estimate HR (95%)	p value	Heterogeneity		Meta-regression		
				I^2^ (%)	p value	tau^2^	Adj R^2^ (%)	p value
**Treatment method**						0.048	-6.76	0.378
Surgery	5	1.80(1.44–2.23)	<0.01	72.2	0.006			
Non- surgery	1	1.35(1.005–1.81)						
**Year**						0.058	-28.15	0.551
>2021	5	1.79(1.40–2.30)	<0.01	70.4	0.009			
<2021	1	1.49(1.30–1.70)						
Sample size						0.053	-17.32	0.541
>1000	3	1.54(1.38–1.71)	<0.01	15.3	0.307			
<1000	3	1.88(1.21–2.93)	0.005	81.7	0.004			
**Design type**						0	100	0.08
Multiple-center	4	1.51(1.37–1.67)	<0.01	1	0.387			
Single-center	2	2.24(1.40–3.60)	0.001	73.2	0.053			

### Sensitivity analysis

**Fig 5:**
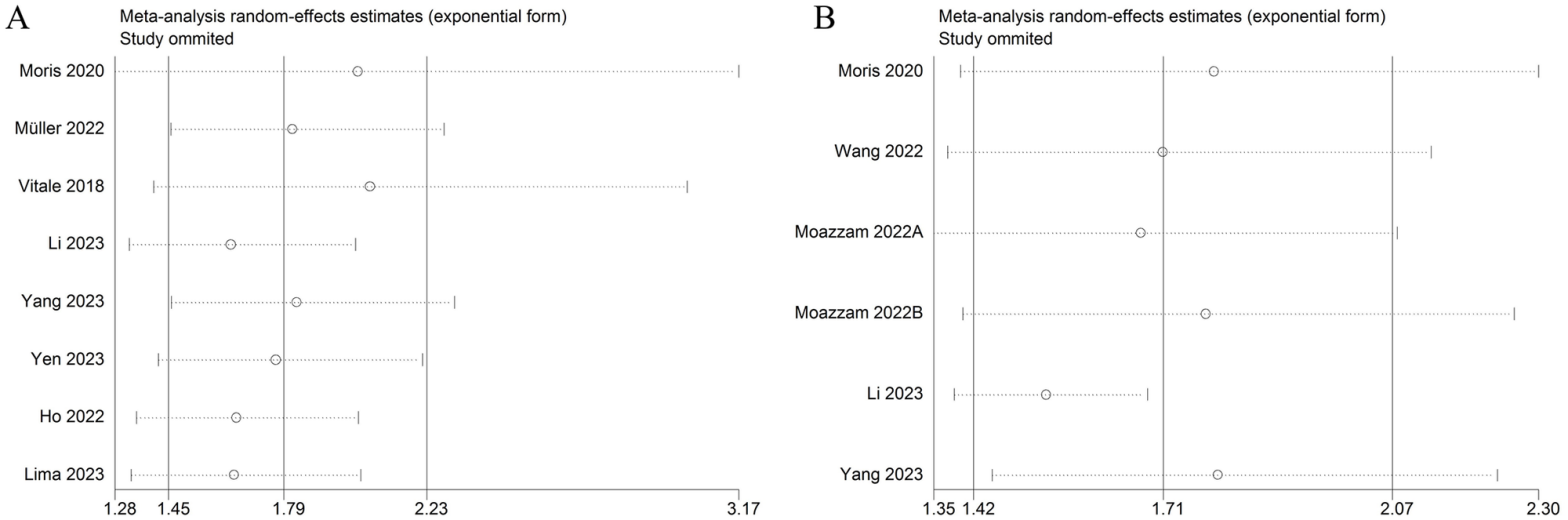
Sensitivity analysis. (A) Sensitivity analysis for OS. (B) Sensitivity analysis for RFS/PFS.

It was an important step in meta-analysis to assess the reliability of the outcomes. By excluding a study once and recalculating the pooled effect estimate, sensitivity analysis could determine whether the overall result was heavily influenced by any single study. In the study, the outcomes for OS and RFS/RFS indicated that the conclusions of the meta-analysis were not significantly affected (Fig 5).

### Publication bias

**Fig 6:**
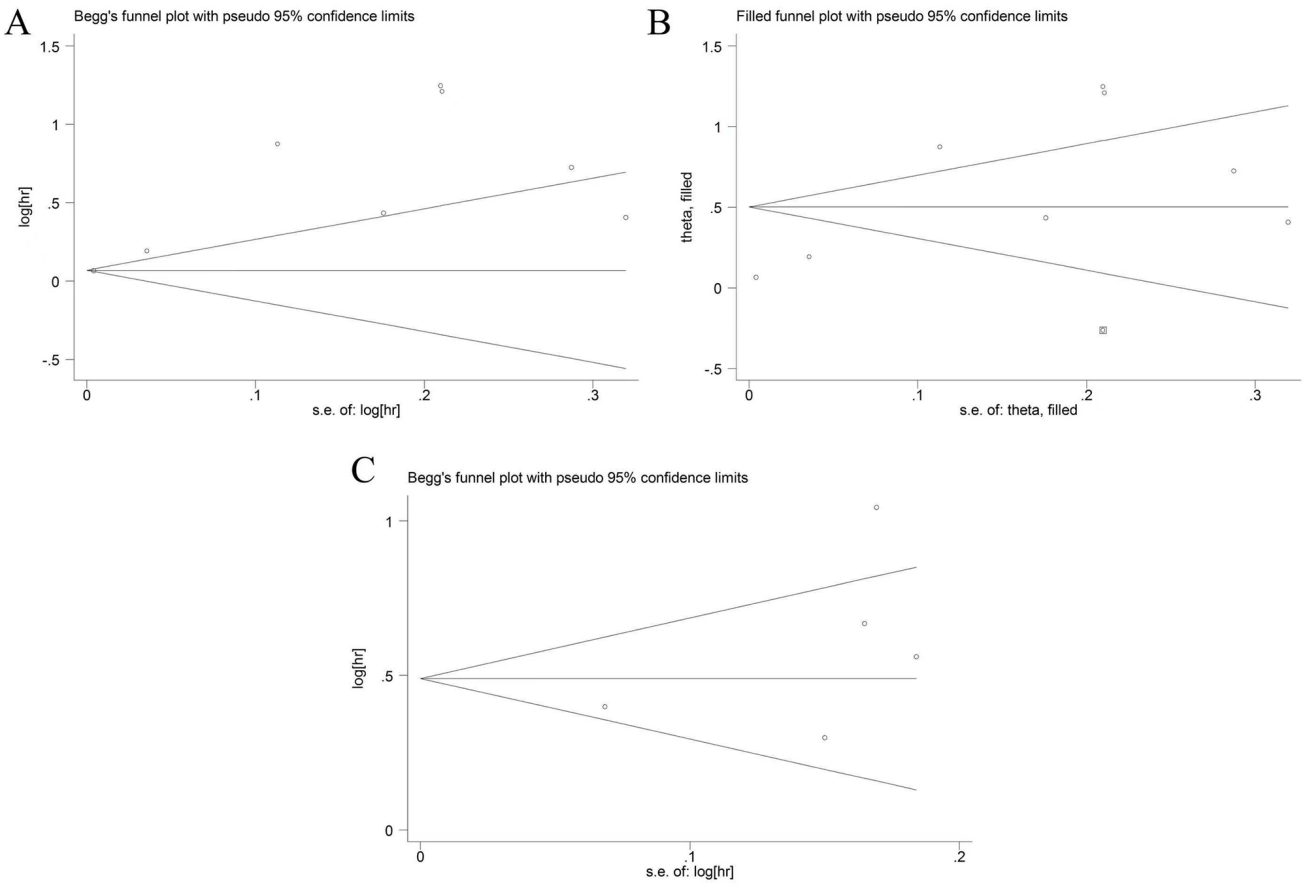
Publication bias. (A) Publication bias for OS. (B) The trim-and-fill method test the OS data. (C) Publication bias for RFS/PFS.

To assess potential publication bias, we conducted the Begg’s test and Egger’s test. The p values of Begg’s test and Egger’s test for OS were 0.902 and 0.004 (Fig 6A), respectively. Although the p-values did not exceed the 0.05 for Begg’s test, significant bias was discovered by Egger’s test. To address this, we performed the trim-and-fill method to explorer the publication bias. The combined results were (1.651, 95%CI:1.346–2.024), indicating that the outcomes were not impacted by the publication bias (Fig 6B). The p-values of Begg’s test and Egger’s test for RFS/PFS were 0.133 and 0.239, indicating that no publication bias were observed (Fig 6C).

## Discussion

Many staging systems have been established based on tumor burden. They are used to stratify the survival of tumor patients and guide treatment. The Barcelona Clinic Liver Cancer (BCLC) has been widely recognized since it is established [25]. But, BCLC is too strict on the selection of patients who can obtain liver resection. It is easy to classify some patients who may obtain liver resection as those who cannot accept hepatectomy [26]. Other scoring systems, such as Japan Integrated Staging (JIS), Cancer of the Liver Italian Program (CLIP) and Metroticket model are not widely used due to its small application scope or complex calculation [27–29]. Accurate prognostic stratification is essential for preoperative assessment of patients. TBS is a novel comprehensive index that integrates tumor sizes with number to evaluate the integral tumor burden. However, the use of TBS in HCC patients remained controversial.

To the best of our knowledge, this investigation was the first meta-analysis to comprehensively analyze the survival value of TBS in HCC patients. The primary findings showed that high TBS was significantly connected with adverse OS and RFS/PFS. Specifically, the results of subgroup analysis showed that TBS had prognostic value in HCC almost independent of treatment methods, sample size, publication year and design type. Few of the included studies involved immunotherapy, targeted therapy or transcatheter arterial chemoembolization, the potential value of TBS in HCC patients receiving these therapies need further be explored.

Evaluation of tumor burden usually involves the tumor size and the tumor number. However, it is difficult to differentiate the survival outcomes of patients with different tumor sizes and numbers. To better forecast the prognosis of tumor patients, it is necessary to convert the dichotomous variables of tumor size and number to continuous variables. TBS is calculated from tumor size and tumor number using the pythagorean theorem. TBS is easy to be calculated, and we need only consider the maximum tumor diameter and tumor number. TBS was first successfully used in patients with colorectal liver metastases and was further demonstrated in cholangiocarcinoma and pancreatic cancer [7, 30, 31]. Tsilimigras et al introduced TBS for the first time in HCC patients undergoing hepatectomy [32]. They found that the survival outcome of HCC patients fluctuated with BCLC stage, but were largely dependent on TBS. Then, Endo et al constructed a model using TBS, AFP, neutrophil-lymphocyte ratio, albumin, gamma-glutamyl transpeptides and vascular involvement, and found that the model was more effective than BCLC in predicting 5-year OS in HCC patients [33]. Lima et al used TBS, AFP and Child-Pugh classification to construct a risk stratification model, which had higher predictive value than BCLC staging [13]. In addition, the model constructed by TBS combined with other inflammatory indicators, such as albumin–bilirubin grade and neutrophil-lymphocyte ratio, was also found to have a higher prediction performance than BCLC and TNM stage [11, 13]. Combined with our meta-analysis, we suggested that TBS was a promising and useful tool to stratify HCC patients. We could use it alone or i combination with other indicators in practical clinical applications.

The investigation had several defections. Firstly, all incorporated publications were retrospective studies, selection bias could not be entirely avoided. Secondly, high heterogeneity may affect the prognostic value of TBS in HCC. Thirdly, the numbers of incorporated studies incorporated in the analysis were limited. Finally, we did not compare TBS with other scoring systems.

There were also some advantages worth noting. Our meta-analysis fully confirmed the prognostic significance of TBS in HCC. In addition, sensitivity analysis, trim-and-fill method, Begg’s test and Egger’s test proved that the outcomes were stable and reliable.

## Conclusion

High TBS was associated with inferior survival outcomes in HCC patients. TBS can be used as a reliable prognostic indicator for HCC. More studies were needed to directly compare the merits and demerits of TBS with other scoring systems before clinical application.

## Supporting information

S1 Checklist PRISMA 2020 checklist. (DOC)

S1 Fig Flow diagram. (DOC)

S1 Table Search strategy. (DOCX)
