# Supplementary material for: High tumor burden score indicated the unfavorable prognosis in patients with hepatocellular carcinoma: A meta-analysis
Source: PLoS One. 2024 Aug 8;19(8):e0308570. doi: 10.1371/journal.pone.0308570 (PMC11309382; doi:10.1371/journal.pone.0308570)
Supplement: S1 Fig — (DOC) [file pone.0308570.s002.doc]

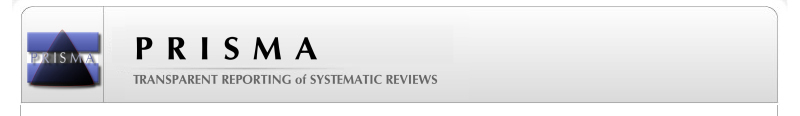
**PRISMA 2009 Flow Diagram**

**Screening**

**Included**

**Eligibility**

**Identification**

Records identified through database searching
(n = 223)

Additional records identified through other sources
(n = 0 )

Records after duplicates removed
(n = 110)

Records screened
(n = 113)

Records excluded
(n =76)

37 articles included in qualitative synthesis

10 studies included in the meta-analysis

Records excluded
(n =27)
