# Supplementary material for: High tumor burden score indicated the unfavorable prognosis in patients with hepatocellular carcinoma: A meta-analysis
Source: PLoS One. 2024 Aug 8;19(8):e0308570. doi: 10.1371/journal.pone.0308570 (PMC11309382; doi:10.1371/journal.pone.0308570)
Supplement: S1 Table — (DOCX) [file pone.0308570.s003.docx]

**Supplementary Table 1.** Search strategy

| Database | Pubmed |
| --- | --- |
| Search | (“tumor burden score”[All Fields]) and (((((hepatocellular carcinoma[All Fields]) OR (hepatic carcinoma[All Fields])) OR (hepatoma[All Fields])) OR (liver cancer[All Fields])) OR (HCC[All Fields])) |
| Results | 57 |
| Database | Web of science |
| Search | (“tumor burden score*”) AND (“hepatocellular carcinoma*”OR “hepatic carcinoma*” OR “bile duct cancer*” OR “hepatomar*” OR “liver cancer*” OR “HCC*” ) |
| Results | 68 |
| Database | Embase |
| Search | ('tumor burden score'/exp OR 'tumor burden score') AND ('hepatocellular carcinoma'/exp OR 'hepatocellular carcinoma' OR (hepatocellular AND ('carcinoma'/exp OR carcinoma)) OR 'hepatic carcinoma'/exp OR 'hepatic carcinoma' OR (hepatic AND ('carcinoma'/exp OR carcinoma)) OR 'hepatoma'/exp OR hepatoma OR 'liver cancer'/exp OR 'liver cancer' OR (('liver'/exp OR liver) AND ('cancer'/exp OR cancer)) OR hcc) |
| Results | 98 |
